# Replication Control of Human Telomere G-Quadruplex DNA by G-Quadruplex Ligands Dependent on Solution Environment

**DOI:** 10.3390/life12040553

**Published:** 2022-04-07

**Authors:** Shuntaro Takahashi, Sudipta Bhowmik, Shinobu Sato, Shigeori Takenaka, Naoki Sugimoto

**Affiliations:** 1 FIBER (Frontier Institute for Biomolecular Engineering Research), Konan University, Kobe 650-0047, Japan; shtakaha@konan-u.ac.jp; 2 Department of Biophysics, Molecular Biology & Bioinformatics, University of Calcutta, Kolkata 700009, India; sbbmbg@caluniv.ac.in; 3 Department of Applied Chemistry, Kyushu Institute of Technology, Fukuoka 804-8550, Japan; shinobu@che.kyutech.ac.jp (S.S.); shige@che.kyutech.ac.jp (S.T.); 4 FIRST (Graduate School of Frontiers of Innovative Research in Science and Technology), Konan University, Kobe 650-0047, Japan

**Keywords:** G-quadruplex, replication, thermodynamics, ligand, topology

## Abstract

The human telomere region is known to contain guanine-rich repeats and form a guanine-quadruplex (G4) structure. As telomeres play a role in the regulation of cancer progression, ligands that specifically bind and stabilize G4 have potential therapeutic applications. However, as the human telomere sequence can form G4 with various topologies due to direct interaction by ligands and indirect interaction by the solution environment, it is of great interest to study the topology-dependent control of replication by ligands. In the present study, a DNA replication assay of a template with a human telomere G4 sequence in the presence of various ligands was performed. Cyclic naphthalene diimides (cNDI1 and cNDI2) efficiently increased the replication stall of the template DNA at G4 with an anti-parallel topology. This inhibition was stability-dependent and topology-selective, as the replication of templates with hybrid or parallel G4 structures was not affected by the cNDI and cNDI2. Moreover, the G4 ligand fisetin repressed replication with selectivity for anti-parallel and hybrid G4 structures without stabilization. Finally, the method used, referred to as quantitative study of topology-dependent replication (QSTR), was adopted to evaluate the correlation between the replication kinetics and the stability of G4. Compared to previous results obtained using a modified human telomere sequence, the relationship between the stability of G4 and the effect on the topology-dependent replication varied. Our results suggest that native human telomere G4 is more flexible than the modified sequence for interacting with ligands. These findings indicate that the modification of the human telomeric sequence forces G4 to rigidly form a specific structure of G4, which can restrict the change in topology-dependent replication by some ligands.

## 1. Introduction

DNAs containing tandem repeats of guanine form guanine-quadruplexes (G4s) comprising guanine quartets linked via Hoogsteen hydrogen bonds. Telomeres contain approximately 100 repeats of T_2_AG_3_, which can form G4 structures. As telomerase binds to these regions to elongate telomeres, G4 formation in telomeres can inhibit the binding and catalysis of telomerase [1]. G4 structures perturb replication [2,3] and transcription, both in vitro and in vivo [4,5]. Previous studies have suggested that the efficiency of the inhibition of these reactions by G4 formation depends not only on the structural stability but also on the topology of G4 formed in the template DNA and RNA [2,4]. As there are more than 300,000 G4-forming potential regions in the human genome [6], the development of ligands that selectively tune the formation of a specific G4 and regulate replication is highly valuable for therapeutic treatment [3,7].

**Figure 1:**
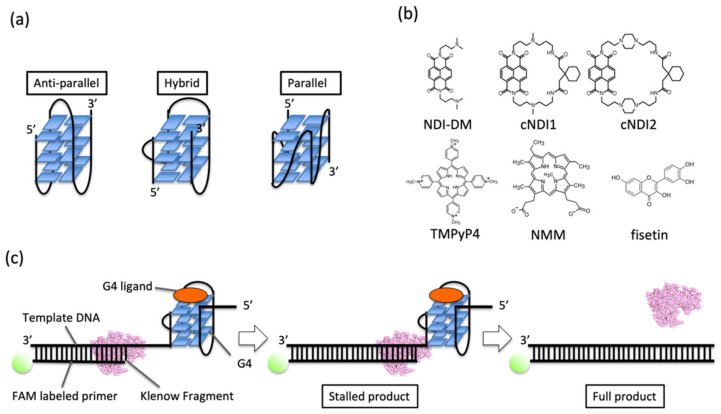
(**a**) Images of G4 topologies. (**b**) Ligands tested in this study. NDI-DM is a non-cyclic naphthalene diimide (NDI) derivative. cNDI1 and cNDI2 are based on NDI-DM and modified with 2,2’-(cyclohexane-1,1-diyl)diacetic acid with linkers of different lengths. Fisetin is a natural product. *Meso*-tetrakis-(*N*-methyl-4-pyridyl)-porphyrin TMPyP4 and *N*-methyl mesoporphyrin IX (NMM) are conventional G4 ligands. (**c**) Schematic illustration of the replication assay in this study.

Although G4 topologies with antiparallel, hybrid, and parallel strands (Figure 1a) have been characterized, only certain G4 structures can be targeted to regulate gene expression. The major interaction between G4 and the ligand is the stacking interaction on the G-quartet [8]. As the G-quartet is a common motif in any type of G4 topology, such G4 ligands cannot distinguish a particular G4 structure. G4 structures differ in their sequence, length, and the configuration of the loop regions. In cells, G4 binding proteins possibly recognize differences in G4 structures [9], but only a few ligands are available to discriminate G4 topologies. These ligands can interact with the loops or grooves of G4 and G-quartets [10,11,12]. Replication of the telomeric region should efficiently occur to maintain the biological roles of telomeres in cells [13], which represents a promising target for cancer therapy [14]. As replication can be influenced by the G4 topology on the DNA template [2], an index for ligand development that shows topology-specific binding and function is highly required.

DNA energetics is fundamental to understanding the stability of DNA structures as well as reaction-related DNA metabolism, such as DNA damage recognition, repair, and replication. Prof Kenneth J. Breslauer, who is the pioneer of this research area, demonstrated a significant relationship between the energetic values of DNA stability and replication efficiency and fidelity in a predictable sequence-dependent manner [15]. Therefore, quantitative analysis of the stability of DNA structures and replication efficiency is fundamental. We previously studied G4 replication in the presence of G4 ligands along a template DNA including a modified sequence of the human telomeric G4-forming sequence (5’-TTGGGTTAGGGTTAGGGTTAGGG-3’), which was different from the native sequence (5’-TTAGGGTTAGGGTTAGGGTTAGGGA-3’) [16]. The modified sequence was developed for NMR measurements to obtain clear spectra. As sequence modification can cause changes in the stability and topology of the G4 structure of human telomeres, the G4-topology-dependent replication of human telomeres should also be affected by sequence modification, although there has been no demonstration. Here, replication of DNA containing a native G4 sequence derived from human telomeres was investigated in the presence of various G4 ligands under different buffer conditions [17]. As shown here, it was found that the replication stall on G4 depended not only on the stability of the G4-ligand complex but also on the manner of interaction of the ligand depending on the G4 topology. The results of this study provide quantitative data showing that the interaction between ligands and G4 controls the energetic barrier of the replicating intermediate, resulting in different unwinding kinetics of G4. Our quantitative study of topology-dependent replication will facilitate drug design to efficiently control gene expression when a mutation occurs in the G4 sequence.

## 2. Materials and Methods

**Materials.** The deoxynucleoside triphosphates (dNTPs) were purchased from Takara Bio (Shiga, Japan). NDI-DM, cNDI1, and cNDI2 previously described [18]. All other ligands are purchased and prepared as described [16].

**Oligonucleotides.** Short DNA for the thermodynamics assay, primer DNA labeled with fluorescein amidite, and template DNA purified using high-performance liquid chromatography (HPLC) were purchased from Japan Bio Service. All of the DNA sequences used in this study are listed in Table S1.

**Replication assay.** For the polymerase, the Klenow fragment exo- (KF exo-) was prepared and used as previously reported [16]. Primer and template DNA were mixed and annealed in the buffer used in the replication reaction: 10 mM Tris-hydrochloric acid (HCl) (pH 7.5), 8 mM magnesium chloride (MgCl_2_), 1 μM DNA, 10 µM each ligand (the concentration of fisetin was 50 µM to facilitate efficient replication stall), and 250 μM dNTPs. Potassium chloride (KCl) and poly(ethylene glycol200) (PEG200) were added as indicated. After preparing the solution, the mixtures were incubated at 37 °C for 5 min followed by the addition of 1 µM KF exo- to initiate the reaction. The reactants were collected at each time point and added to a solution containing 10 mM EDTA and 80 wt% formamide. After running urea-polyacrylamide gel electrophoresis (PAGE) containing 8 M urea at 70 °C for 1 h at 200 V in TBE buffer, the gel images were captured using a Fluoreimager FLA-5100 (Fujifilm, Tokyo, Japan) before and after staining with SYBR Gold (Thermo Fisher Scientific, Tokyo, Japan). The intensities of the bands were analyzed using NIH ImageJ software. The amount of full-length product (*P*) was quantified by calculating the ratio of the intensity of the full-length product band to that of all bands. A kinetic model was applied to a two-step sequential model.

(1) $$P_{0}\overset{k_{s}}{\to }P_{S}\overset{k_{f}}{\to }P_{F}$$

where *P*_0_ is the starting state of the reaction, *P*_s_ is the state immediately after dissolving the stall, *P_F_* is the state after replication of the full-length product is completed, *k*_s_ (min^−1^) is the rate constant from the start of the reaction to dissolving the stall, and *k_f_* (min^−1^) is the rate constant from dissolving the stall to complete synthesis of the full-length product. The kinetic analyses were performed as previously described [16].

**UV melting assay.** The solution used for the ultra violet (UV) melting assay included 5 µM (T_2_AG_3_)_4_ and 10 μM of each ligand. For fisetin, a 50 µM of the solution was used. All samples were dissolved in the solution containing the buffer used in the replication assay except for dNTPs. Melting and thermodynamic analyses were performed as previously described [16].

**CD Spectroscopy Measurements.** The sample preparation for CD measurement was same as the UV melting assay, followed by sample annealing. CD measurement were performed using a JASCO J-1500 instrument at 37 °C as previously [16].

## 3. Results

### 3.1. Structure and Stability of Human Telomeric G4 in the Presence of Ligands in a Low K^+^ Ion Concentration

The replication of G4 DNA was studied using the Klenow fragment of DNA polymerase I from *Escherichia coli* that lacks exonuclease activity (KF exo-) in the presence of non-cyclic (NDI-DM) and cyclic naphthalene diimide (NDI) derivatives (cNDI1 and cNDI2) [18], and conventional G4 porphyrin ligands (*meso*-tetrakis-(*N*-methyl-4-pyridyl)-porphyrin (TMPyP4) and *N*-methyl mesoporphyrin IX (NMM) (Figure 1b,c). Fisetin, which binds to the loop regions of G4 [19], was assayed. The template DNA contained a G4-forming sequence (four repeats of the human telomeric DNA sequence (T_2_AG_3_)_4_) downstream of the region complementary to the primer-binding site (Table S1). This sequence can form various topologies, depending on the solution conditions [17].

**Figure 2:**
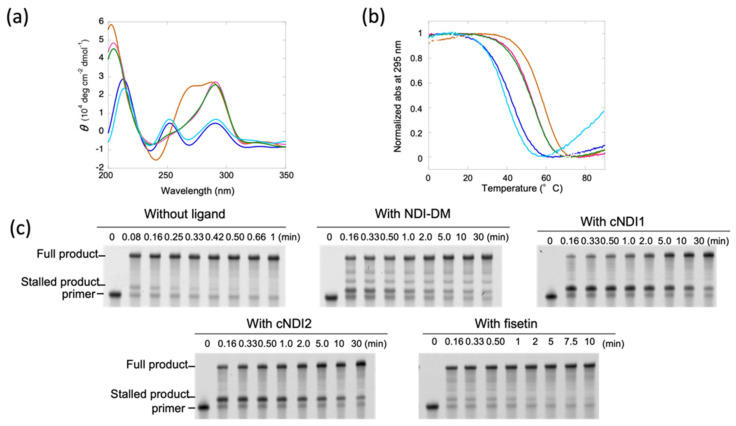
(**a**) CD spectra at 37 °C and (**b**) ultra violet (UV) melting curves of 5 µM (T_2_AG_3_)_4_ in the absence (blue) and presence of 10 μM non-cyclic naphthalene diimide (NDI)-DM (pink), cyclic (**c**) NDI1 (brown), cNDI2 (green), or 50 µM fisetin (light blue). The buffer condition was 10 mM Tris-hydrochloric acid (HCl) (pH 7.5), 1 mM potassium chloride (KCl), and 8 mM magnesium chloride (MgCl_2_). (**c**) Polyacrylamide gel electrophoresis (PAGE) images of aliquots of replication reactions of 1 µM (T_2_AG_3_)_4_ template DNA in the absence or presence of 10 µM NDI-DM, cNDI1, cNDI2, or 50 µM fisetin. The reaction was carried out with 1 µM KF exo- and 250 µM deoxynucleoside triphosphates (dNTPs) at 37 °C. Aliquots were taken at time intervals that depended on the ligand.

The topology of G4 DNA from human telomeric sequence is dependent on the concentration of K^+^ ion; for example, the oligonucleotide (T_2_AG_3_)_4_ forms an anti-parallel structure at a low concentration of K^+^ ions [20]. In the presence of 1 mM KCl in a buffer containing 10 mM Tris HCl (pH 7.5) and 8 mM MgCl_2_, the circular dichroism (CD) spectra of 5 µM (T_2_AG_3_)_4_ in the absence of ligands at 37 °C showed a large positive peak around 295 nm (Figure 2a), indicative of an anti-parallel conformation [11]. As the stability of G4 is low in this buffer condition, the spectrum shows the mixture of the spectrum with the coil form, which showed peaks at 220 nm and 255 nm. We measured the CD spectra of the DNA with 10 µM NDI ligands. Except for the spectrum in the presence of cNDI1 showing a hybrid topology with peaks at 265 and 295 nm, all indicated that G4s in these conditions adopted an anti-parallel conformation (Figure 2a) [11]. The spectrum in the presence of fisetin also indicated an antiparallel topology with the mixture of coil form due to low stability of G4. 

Next, UV melting measurements were performed to quantitatively analyze the thermodynamic stability of G4 structures in the absence and presence of ligands (Figure 2b). In the absence of ligands, the melting temperature was 42.7 °C, and the thermodynamic stability at 37 °C (−∆*G*°_37_) of the G4 structures, which was used to quantitatively compare the stability, was 0.6 kcal mol^−1^ (Table 1). On the other hand, −∆*G*°_37_ in the presence of NDI-DM was 1.9 kcal mol^−1^, indicating that the structure was stabilized by the ligand. For the cases of cyclic NDI ligands, −∆*G*°_37_ was 2.5 (cNDI1) and 1.8 (cNDI2) kcal mol^−1^, respectively, which were also larger than those in the absence of ligands. Fisetin slightly destabilized the G4 with a −∆*G*°_37_ of 0.4 kcal mol^−1^ (Table 1).

### 3.2. Relationship between Thermodynamic Stability of the G4 Structure and Its Replication Efficiency in a Low K^+^ Concentration

The replication reaction was performed in the same buffer in the presence of 1 μM KF exo- and 250 μM dNTPs. After 0.08 min (=5 s), the two products were detected by denaturing polyacrylamide gel electrophoresis (PAGE; Figure 2c). The product migrating faster was confirmed to be the fully extended primer after staining in comparison with the mobility of the template DNA (Figure S1). The product migrating slower, which extended only a few nucleotides from the primer (Figure 2c and Figure S1), indicated a stalled product due to its G4 structure. This stalled product disappeared after 0.33 min (=20 s), indicating that replication was stalled to some extent by the G4 structure.

In the presence of NDI-DM, these two replication products were mainly as observed in the absence of the ligand. However, the stalled product was much accumulated more than that without the ligand (Figure 2c), which indicates that NDI-DM stabilized G4 and thus increased the replication stall compared to that without the ligand. The minor bands observed between the bands of the stalled product and full-length product may be derived from replication stall by the nonspecific binding of NDI-DM due to the relatively low specificity for G4 [18]. More stalled products were detected in the presence of cNDI1 and cNDI2 than in the presence of NDI-DM (Figure 2c). Contrary to our expectations based on the melting results, which showed that the −∆*G*°_37_ values were not significantly different in the presence of NDI-DM and cNDI2, although cNDI1 slightly stabilized the (T_2_AG_3_)_4_ structure relative to NDI-DM, more significant stalling of the polymerase enzyme was induced by cNDI1 and cNDI2 than NDI-DM. Replication was also analyzed in the presence of TMPyP4 and NMM as conventional G4 ligands, and found almost no polymerase stalling (Figure S2a,b), likely due to the fact that TMPyP4 and NMM do not bind well to anti-parallel G4 [21]. Therefore, cNDIs may stall replication effectively due to the interaction between the cyclic moieties and G4 loop regions. Moreover, in the presence of fisetin, a small amount of the stalled product was observed.

To compare the replication kinetics and thermodynamic stability of (T_2_AG_3_)_4_, we determined the kinetics of the replication of the full-length products, as previously reported (Figure S3) [2]. A two-step sequential model consisting of the stall in replication was assumed, which included the prior replication to the stall position, and generating a full-length product after resolving the replication stall. The rate constants (*k*_s_) at 37 °C before releasing the replication stall of KF exo- were 4.4 min^−1^ (without ligand), 0.68 min^−1^ (with NDI-DM), 0.24 min^−1^ (with cNDI1), and 0.14 min^−1^ (with cNDI2) (Table 1). The rate-limiting step of these reactions was the resolution of G4 since the rate constants *k*_f_ for replication after dissolving the stall was much larger than *k*_s_ (Table 1). For fisetin, the *k*_s_ values (0.60 min^−1^) were also smaller than the *k*_f_ values. Therefore, stall of replication is the rate-limiting step of the reaction. The *k*_f_ values were similar for all of the ligands, and the relatively large difference between the *k*_f_ without ligand and that with ligand indicated a decrease in the processivity of the single-stranded region downstream of the G4 structure owing to the effect of nonspecific binding of ligands to the template DNA.

The *k*_s_ values indicated that the cNDI ligands inhibited the replication of the (T_2_AG_3_)_4_ template more effectively than the parent compound NDI-DM. Each of these NDI compounds has a naphthalene moiety that presumably interacts in a similar manner to a G-quartet; thus, the cyclic moieties of cNDI1 and cNDI2 influence binding to (T_2_AG_3_)_4_. Interestingly, NDI-DM and cNDI2 similarly stabilized the G4 conformation of (T_2_AG_3_)_4_, but the efficiency of replication repression differed. Furthermore, the *k*_s_ of fisetin was much smaller than that in the absence of ligands, although the stability of the (T_2_AG_3_)_4_ G4 did not increase. These effects on the replication rate that do not correlate with G4 stability imply that the binding mode of the ligand to G4 influences how efficiently the structure is resolved by polymerase.

### 3.3. Quantitative Analysis of G4 Stability and G4 Replication Affected by G4 Ligands

**Figure 3:**
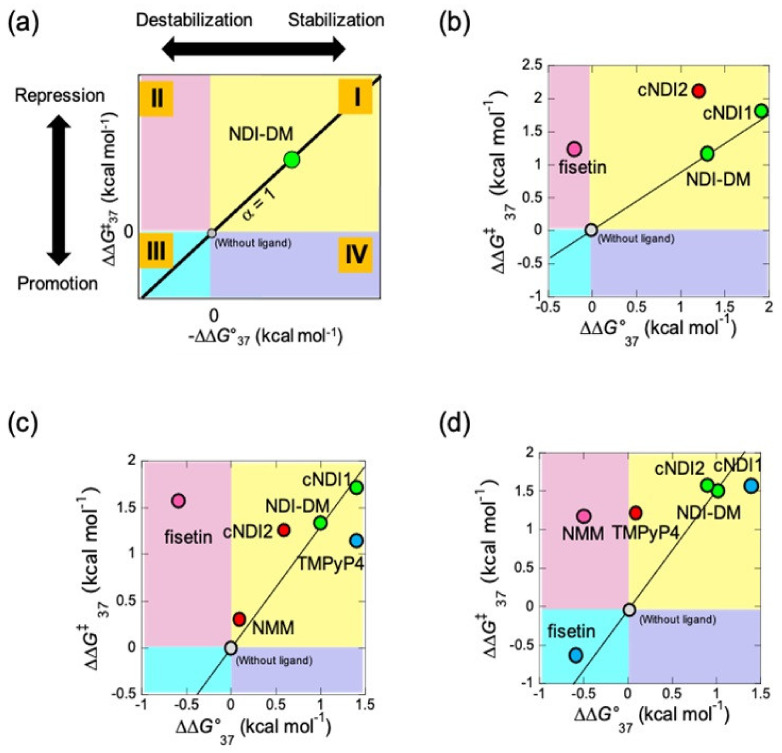
Quantitative study of topology-dependent replication (QSTR) for the native human G4 sequence. (**a**) The scheme of QSTR plot. The different quadrant shows each combination of positive and negative value of −∆∆*G*°_37_ and ∆∆*G*^‡^_37_ (I–IV). The value α, which indicates the ratio of ∆∆*G*^‡^_37_/(−∆∆*G*°_37_) of the ligand of interest to that of the NDI-DM. Reprinted with permission from [16]. Copyright 2021 American Chemical Society. (**b**–**d**) Obtained QSTR plots in (**b**) 1 mM potassium chloride (KCl) without poly(ethylene glycol200) (PEG200), (**c**) in the presence of 100 mM KCl without PEG200, and (**d**) 1 mM KCl with 20 wt% PEG200. The degree of α was shown in different colors in the plot (α ≈ 1; green, α > 1; red, 0 < α < 1; blue, and α < 0; pink).

To quantitatively analyze the effects of the ligand on replication through G4s of different topologies, a method was developed for novel categorization of G4 ligands named quantitative study of topology-dependent replication (QSTR) to analyze the phase diagram of the stability of G4 vs. activation energy of G4 replication, which we established previously (Figure 3) [2,16,22]. Briefly, QSTR categorizes and quantitatively characterizes the effect of ligands on stalling at G4. In the analysis, it was demonstrated that the plot between the −∆∆*G*°_37_ values (−∆*G*°_37_ in the presence of ligand—(−∆*G*°_37_ in the absence of ligand)) and ∆∆*G*^‡^_37_ values (∆*G*^‡^_37_ is the activation free energy of replication of G4 at 37 °C). Furthermore, the slope (∆∆*G*^‡^_37_/(−∆∆*G*°_37_)) of the x-y surface of the ligand of interest, *S*_i_, was divided by that of the standard ligand *S*. A comparison of the slopes represents the obtained value α, that is, *S*_i_/*S*. Here, the standard is NDI-DM.

To visualize the relationship between the stability of G4 and replication kinetics, the plots were categorized into upper, lower, and right or left quadrants in different colors (groups I, II, III, and IV are yellow, green, light blue, and purple, respectively) (Figure 3A). Ligands in groups I and III changed the unfolding kinetics of G4, which resulted in replication control. However, ligands in groups II and IV exhibit control over replication that is independent of the effect on the stability of G4. The categorized ligands were also arranged in in category i (α ≈ 1), ii (α > 1 indicating that the reaction is ∆∆*G*^‡^_37_ dependent), and iii (α < 1 indicating that the reaction is ∆∆*G*°_37_ dependent) in addition to iv (α < 0). The QSTR of the data for 1 mM KCl without PEG200 (Figure 3B and Table 2) indicated that cNDI1 was group I-i, which means that cNDI1 has the same mechanism of replication stall (α ≈ 1) as that of NDI-DM. The cNDI2 data corresponded to relationship (I-ii), suggesting that these ligands stabilize G4 and inhibit the replicating polymerase differently and more efficiently than NDI-DM. The fisetin data also deviated and represented relationship (II-iv), indicating that the ligand repressed the replication of G4 with a smaller ∆*G*^‡^ than that of NDI-DM.

### 3.4. Categorization of Ligands by Quantitative Study of Topology-Dependent Replication (QSTR) Analysis

Regarding 100 mM KCl without PEG200 condition, from the CD spectrum at 37 °C, the native human G4 sequence showed a hybrid topology, as previously reported [23], whereas all NDI ligands induced an anti-parallel topology, except for cNDI1 (Figure S4a). The spectrum in the presence of cNDI1 differed slightly from that of the others, suggesting a slightly different structural change forming the hybrid topology. For TMPyP4 and NMM, the spectra indicated a parallel topology, whereas fisetin had an antiparallel topology (Figures S2g and S4a).

At 100 mM KCl, stability and replication kinetics parameters were obtained (Figures S2i and S4b,c, Table 1). −∆∆*G*°_37_ vs. ∆∆*G*^‡^_37_ for NDI-DM, and cNDI1 fell in the QSTR categories I-i (Figure 3c and Table 2). Under these conditions, the G4 of (T_2_AG_3_)_4_ formed a hybrid topology in the presence of these ligands. Thus, QSTR indicates that these ligands affected the replication stall through the same mechanism as the standard. cNDI2 and NMM were categorized as I-ii, but the difference in the relative activation energy suggested that NMM repressed replication more efficiently than cNDI2. TMPyP4 was categorized as I-iii, indicating that replication was repressed less efficiently than in the case of other ligands due to a small effect on the intermediate formed during replication. Fisetin was categorized as into II-vi, as was the case with 1 mM KCl without PEG200, suggesting that the effect of fisetin on replication stalling was not influenced by the topology of G4 but that the inhibition mechanism may be shared in each case.

As molecular crowding can change G4 topology [17], the QSTR analysis was also performed in the presence of 20 wt% poly(ethylene glycol) 200 (PEG200; average molecular weight 200). In 20 wt% PEG200, 10 mM Tris-HCl (pH 7.5), 8 mM MgCl_2_, and 1 mM KCl at 37 °C, the CD spectrum of (T_2_AG_3_)_4_ indicated a hybrid topology (Figure S4d). Despite the low concentration of K^+^, PEG200 partially induced a structural change from the antiparallel to the hybrid form. The spectra with cNDI1, cNDI2, and fisetin were indicative of similar CD patterns, although NDI-DM showed an anti-parallel topology (Figure S4d). TMPyP4 and NMM also exhibited spectra with parallel topologies (Figure S2h).

Based on the thermodynamic and replication assays in 1 mM KCl and 20 wt% PEG200 (Figures S2j and S4e,f, Table 1), QSTR analysis indicated that α the of NDI-DM, cNDI1, and cNDI2 data were almost 1, resulting in QSTR categories I-i (Figure 3d and Table 2). Fisetin was categorized as III-iii, indicating that fisetin destabilized G4 and promoted replication due to the acceleration of the kinetics of G4 unfolding. TMPyP4 was categorized into I-ii, which may depend on how the ligand induces the parallel topology. The QSTR category for NMM was II-iv, suggesting that the energy barrier for the replicating intermediate influences replication kinetics. All of the plots of NDI ligands may fall into category I-i with the use of TMPyP4 or NMM if all NDI ligands and fisetin bind to the perfectly formed parallel topology. In summary, the interaction of G4 ligands with G4 structures can vary under different conditions, which causes the regulation of G4 replication in a topology-dependent manner.

## 4. Discussion

In this study, QSTR analysis of G4 replication along the template DNA was performed, including a native human telomere sequence (5’-TTAGGGTTAGGGTTAGGGTTAGGG-3’). As shown in Table 2, the results obtained in this study were compared with those obtained for the modified sequence (5’-TTGGGTTAGGGTTAGGGTTAGGGA-3’). Some QSTR categories of the native sequences were the same as those of the modified sequences. For example, cNDI2 in 1 or 100 mM KCl without PEG200 showed the same QSTR categories. Contrastingly, other QSTR categories differed from those of the modified sequence. In the case of cNDI1, the QSTR categories for the native sequence were I-i in all solution conditions and I-ii for the modified sequence. In a previous study, an interaction between the loop region of the G4 and ligand moiety of cNDI1 and cNDI2 was identified, which caused different QSTR categories from the standard ligand NDI-DM [16]. As NDI-DM has no cyclic moiety, the interaction between the ligand and the loop region should be small. The QSTR category I-i of cNDI1 indicates that the unwinding mechanism of replication is similar to NDI-DM. Therefore, the results of this study suggest that the interaction of cNDI1 with the loop region of G4 was not strong and had no significant effect on the efficient replication stall. For cNDI2, the QSTR category in the solution containing 1 mM KCl and 20 wt% PEG200 was I-i, which was different from that for the modified sequence. This could be due to topology change, which lowered the interaction between the loop region of G4 and cNDI2. A similar change was observed in the case of fisetin in the solution of 100 mM KCl without PEG200, which showed different QSTR categories compared with the native and modified sequences owing to the topology change of G4. The authors hypothesize that these behaviors of the native sequence are derived from the flexibility of the structure. Considering the availability of structural analysis, the modified sequence has only a small amount of structural polymorphism and is beneficial for structural analyses such as NMR measurements [16]. Thus, the rigid structure provides a specific interaction geometry for the ligands and causes a topology-dependent replication stall. On the other hand, the G4 structure from the native sequence was flexible and formed a different structure from the ligand-G4 complex. Therefore, the effect of topology-dependent replication was different from that of the ligand structure. In truth, we could assign NMM and TMPyP4 to the QSTR categories in the case of the native sequence, whereas NMM and TMPyP4 were not enough to stabilize the G4 of the modified sequence in most cases. QSTR provides information on the potential for replication control along different template sequences. From the view point of the specific interaction derived from the difference of the sequence, there are two possible interactions by the additional adenine in the sequence of the native telomere sequence. One is the stack on the G-quartet, which can differently regulate the topology of G-quadruplex compared with the modified sequence. Another one is the adenine base in the native sequence may differently interact with the ligands compared with the case of the modified sequence. In the anti-parallel and hybrid structure, these interactions happened affect QSTR categories of the NDI ligands as you suggested, since NDI ligands interact each G-quartet of the G-quadruplex and loop regions as well.

In conclusion, it was identified that the regulation of topology-dependent replication by G4 ligands depends on the structural flexibility of G4 owing to sequence modification. These results suggest that naturally occurring modifications, such as methylation and oxidation, can cause perturbation of G4 flexibility and thus, changes in topology-dependent replication of the telomere region, which would be useful for designing ligands under many conditions for treating diseases.

## Figures and Tables

**Table 1:** Thermodynamic parameters for the formation of human native G4 structure and kinetic parameters for G4 replication at 37 °C.

DNA ^a^ with Ligand	Condition ^b^	*T*_m_ (°C) (°C)	−∆*G*°_37_ (kcal mol^−1^)	*k*_s_ (min^−1^) (min^−1^)	*k*_f_ (min^−1^) (min^−1^)
(T_2_AG_3_)_4_	1 mM KCl, 0 wt% PEG200	42.7 ± 0.7	0.6 ± 0.1	4.4 ± 2.2	71 ± 40
(T_2_AG_3_)_4_ + NDI-DM		52.9 ± 1.0	1.9 ± 0.1	0.68 ± 0.01	10 ± 1.5
(T_2_AG_3_)_4_ + cNDI1		58.7 ± 1.0	2.5 ± 0.2	0.24 ± 0.11	8.5 ± 3.0
(T_2_AG_3_)_4_ + cNDI2		52.3 ± 1.8	1.8 ± 0.3	0.14 ± 0.06	9.6 ± 1.3
(T_2_AG_3_)_4_ + fisetin		41.4 ± 0.2	0.4 ± 0.2	0.60 ± 0.04	9.6 ± 2.8
(T_2_AG_3_)_4_	100 mM KCl, 0 wt% PEG200	73.7 ± 0.2	4.0 ± 0.1	0.36 ± 0.08	7.6 ± 1.6
(T_2_AG_3_)_4_ + NDI-DM		76.6 ± 0.2	5.0 ± 0.2	0.042 ± 0.019	1.0 ± 0.3
(T_2_AG_3_)_4_ + cNDI1		79.3 ± 0.9	5.4 ± 0.1	0.023 ± 0.064	0.8 ± 0.1
(T_2_AG_3_)_4_ + cNDI2		76.0 ± 0.2	4.6 ± 0.1	0.047 ± 0.025	1.6 ± 0.4
(T_2_AG_3_)_4_ + TMPyP4		74.8 ± 0.3	5.4 ± 0.3	0.056 ± 0.005	1.3 ± 0.1
(T_2_AG_3_)_4_ + NMM		72.4 ± 0.4	4.1 ± 0.1	0.23 ± 0.03	4.9 ± 0.9
(T_2_AG_3_)_4_ + fisetin		70.6 ± 0.1	3.4 ± 0.1	0.028 ± 0.003	1.3 ± 0.1
(T_2_AG_3_)_4_	1 mM KCl, 20 wt% PEG200	45.4 ± 0.1	1.1 ± 0.1	0.49 ± 0.19	6.3 ± 1.4
(T_2_AG_3_)_4_ + NDI-DM		53.3 ± 0.9	2.1 ± 0.1	0.042 ± 0.001	2.3 ± 0.14
(T_2_AG_3_)_4_ + cNDI1		57.0 ± 1.2	2.5 ± 0.2	0.039 ± 0.008	1.9 ± 0.03
(T_2_AG_3_)_4_ + cNDI2		51.7 ± 1.5	2.0 ± 0.2	0.039 ± 0.009	3.3 ± 0.29
(T_2_AG_3_)_4_ + TMPyP4		47.5 ± 0.4	1.2 ± 0.1	0.069 ± 0.009	2.7 ± 0.1
(T_2_AG_3_)_4_ + NMM		42.7 ± 0.1	0.6 ± 0.1	0.073 ± 0.006	3.7 ± 0.1
(T_2_AG_3_)_4_ + fisetin		40.1 ± 0.2	0.5 ± 0.1	1.4 ± 0.4	14 ± 2.4

^a^ The concentration of DNA was 5 µM. ^b^ The sample solution contained 10 mM Tris-hydrochloric acid (HCl) (pH 7.5), 1 or 100 mM potassium chloride (KCl), and 8 mM magnesium chloride (MgCl_2_) with or without poly(ethylene glycol200) (PEG200).

**Table 2:** G4 topologies of (T_2_AG_3_)_4_ and categories of QSTR.

Condition	Ligand	G4 Topology (T_2_AG_3_)_4_	QSTR Category (T_2_AG_3_)_4_	G4 Topology T_2_(G_3_T_2_A)_3_A ^b^	QSTR Category T_2_(G_3_T_2_A)_3_A _4_ ^b^
1 mM KCl without PEG200	NDI-DM	Anti-parallel	I-i ^a^	Anti-parallel	I-i ^a^
	cNDI1	Hybrid	I-i	Hybrid	I-ii
	cNDI2	Anti-parallel	I-ii	Anti-parallel	I-ii
	fisetin	Anti-parallel	II-iv	n.d.	n.d.
100 mM KCl without PEG200	NDI-DM	Anti-parallel	I-i ^a^	Anti-parallel	I-i ^a^
	cNDI1	Hybrid	I-i	Hybrid	I-ii
	cNDI2	Anti-parallel	I-ii	Anti-parallel	I-ii
	TMPyP4	Parallel	I-iii	n.d.	n.d.
	NMM	Parallel	I-ii	n.d.	n.d.
	fisetin	Anti-parallel	II-iv	Hybrid	III-iii
1 mM KCl and 20 wt% PEG200	NDI-DM	Anti-parallel	I-i ^a^	Anti-parallel	I-i ^a^
	cNDI1	Hybrid	I-i	Hybrid	I-ii
	cNDI2	Hybrid	I-i	Anti-parallel	I-ii
	TMPyP4	Parallel	I-ii	n.d.	n.d.
	NMM	Parallel	II-iv	Hybrid	II-iv
	fisetin	Hybrid	III-iii	n.d.	n.d.

^a^ NDI-DM is a standard ligand. ^b^ Results obtained from our previous study.

## Supplementary Materials

The following are available online at https://www.mdpi.com/article/10.3390/life12040553/s1. Figure S1: Images of gels shown in Figure 2b stained with SYBR Gold reagent, Figure S2: Effect of TMPyP4 and NMM on replication and structure of G4; Figure S3: Time course of full-length product of the replication reaction; Figure S4: Spectroscopic data and replication results in the presence of 100 mM KCl or in the presence of 1 mM KCl and 20 wt% PEG200; Table S1: DNA sequences used in this study; Table S2: QSTR data calculated using the parameters listed in Table 2.
