# Supplementary material for: Replication Control of Human Telomere G-Quadruplex DNA by G-Quadruplex Ligands Dependent on Solution Environment
Source: Life (Basel). 2022 Apr 7;12(4):553. doi: 10.3390/life12040553 (PMC9024748; doi:10.3390/life12040553)
Supplement: Supplementary file 1 [file life-12-00553-s001.zip › life-1669289-supplementary.pdf]

Table S1. DNA sequences used in this study

FAM-labeled primer	5'-FAM-GGGATGATACTAGTGCTTCGGCTTAATACGACTCACTATAGGG-3'
$(T_2AG_3)_4$ template	5'-TTAGGGTTAGGGTTAGGGTTAGGGCGGCCCCTATAGTGAGTCGTATTAAGCC GAAGCACTAGTATCATCCC-3'
$(T_2AG_3)_4$	5'-TTAGGGTTAGGGTTAGGGTTAGGG-3’

Table S2. Quantitative study of topology-dependent replication (QSTR) data calculated using the parameters listed in Table 2

Ligand	Condition[a]	$-\Delta\Delta G^{\circ}_{37}$ (kcal mol^{-1})	$\Delta\Delta G^{\ddagger}_{37}$ (kcal mol^{-1})	S_i [b]	α [c]
NDI-DM	1 mM KCl 0 wt% PEG200	1.3	1.2	0.86	1
cNDI1		1.9	1.8	0.94	1.1
cNDI2		1.2	2.1	1.77	2.0
fisetin		-0.2	1.2	-6.1	-6.9
NDI-DM	100 mM KCl 0 wt% PEG200	1	1.3	1.3	1
cNDI1		1.4	1.7	1.2	0.9
cNDI2		0.6	1.3	2.1	1.6
TMPyP4		1.4	1.1	0.82	0.6
NMM		0.1	0.3	2.8	2.1
fisetin		-0.6	1.6	-2.6	-1.9
NDI-DM	1 mM KCl 20 wt% PEG200	1	1.5	1.5	1
cNDI1		1.4	1.6	1.1	0.7
cNDI2		0.9	1.6	1.7	1.1
TMPyP4		0.1	1.2	12.1	8.0
NMM		-0.5	1.2	-2.3	-1.5
fisetin		-0.6	-0.6	1.1	0.7

[a] All experiments were performed in 10 mM Tris-hydrochloric acid (HCl) (pH 7.5), 1 or 100 mM potassium chloride (KCl), and 8 mM magnesium chloride ($MgCl_2$) at 37 °C with or without poly(ethylene glycol200) (PEG200).

[b] S_i was calculated using the following formula: ($\Delta\Delta G^{\ddagger}_{37}$ for each ligand)/($-\Delta\Delta G^{\circ}_{37}$ for each ligand).

[c] α was calculated using the following formula: Si/S, where S is the S_i of naphthalene diimides (NDI)-DM.

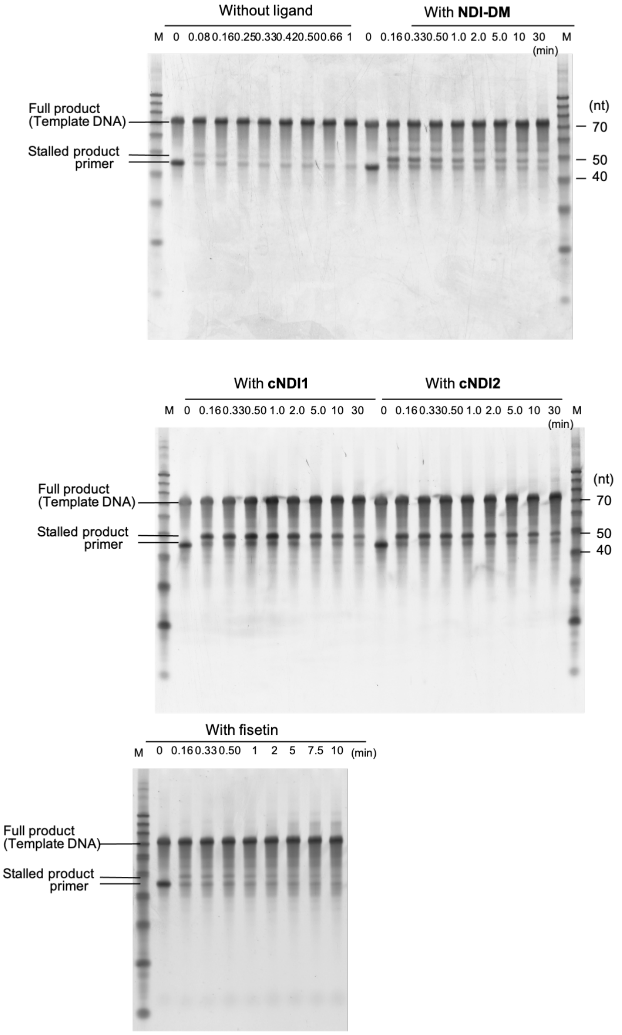

Figure S1. Images of the gels shown in Figure 2b were stained with the SYBR Gold reagent. Fluorescently labeled primers, full-length products, stalled products, and template strands were visible in these images. The molecular weight markers (lane M) show a 10-bp DNA ladder. In each lane, the uppermost band corresponds to the template DNA and the lowest band corresponds to the FAM-labeled primer. The reaction mixture was incubated for the indicated times in the absence or presence of ligand. In the samples incubated with the enzyme, the full-length product band overlapped with that of the template DNA, and the stalled product, which migrated just above the primer, was observed.

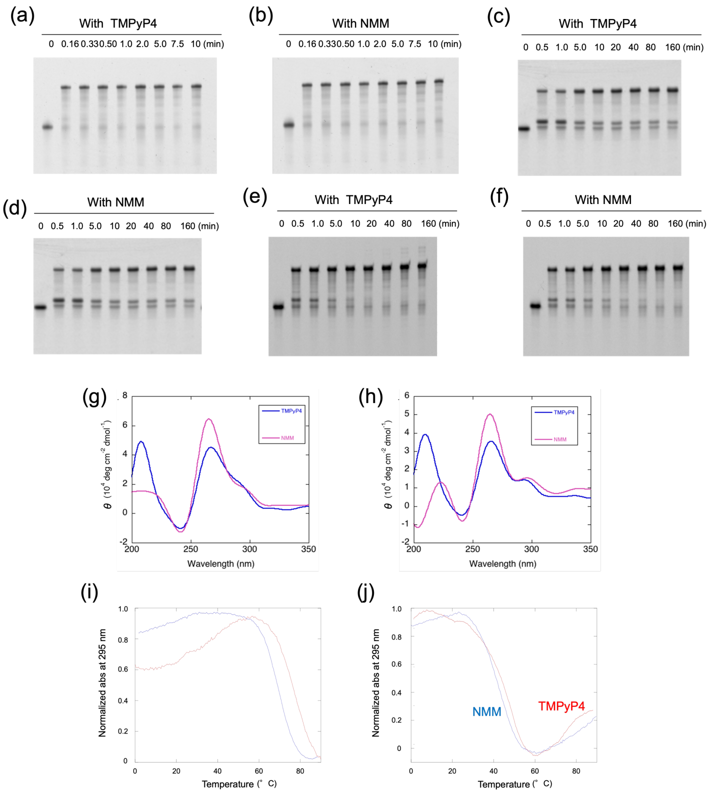

Figure S2. The effect of *meso*-tetrakis-(*N*-methyl-4-pyridyl)-porphyrin (TMPyP4) and *N*-methyl mesoporphyrin IX (NMM) on replication and structure of G4. (a-f) PAGE images of time courses of the replication of 1 μM template DNA containing $(T_2AG_3)_4$ sequence with (a) 10 μM TMPyP4 in the presence of 1 mM potassium chloride (KCl), (b) 10 μM NMM in the presence of 1 mM KCl, (c) 10 μM TMPyP4 in the presence of 100 mM KCl, (d) 10 μM NMM in the presence of 100 mM KCl, (e) 10 μM TMPyP4 in the presence of 1 mM KCl and 20 wt% poly(ethylene glycol200) (PEG200), and (f) 10 μM NMM in the presence of 1 mM KCl and 20 wt% PEG200. The reactions were performed at 37 °C using 1 μM KF and 250 μM deoxynucleoside triphosphates (dNTPs) in 10 mM Tris-hydrochloric acid (HCl) (pH 7.5), 8 mM magnesium chloride ($MgCl_2$) with indicated concentration of KCl and with or without PEG200, as indicated. (g, h) CD spectra of 5 μM $(T_2AG_3)_4$ in the presence of 10 μM TMPyP4 and 10 μM NMM (g) in a solution containing 100 mM KCl and (h) a solution containing 1 mM KCl with 20 wt% PEG200. (i, j) UV melting of 5 μM $(T_2AG_3)_4$ in the presence of 10 μM TMPyP4 and 10 μM NMM (i) in a solution containing 100 mM KCl and (j) a solution containing 1 mM KCl with 20 wt% PEG200.

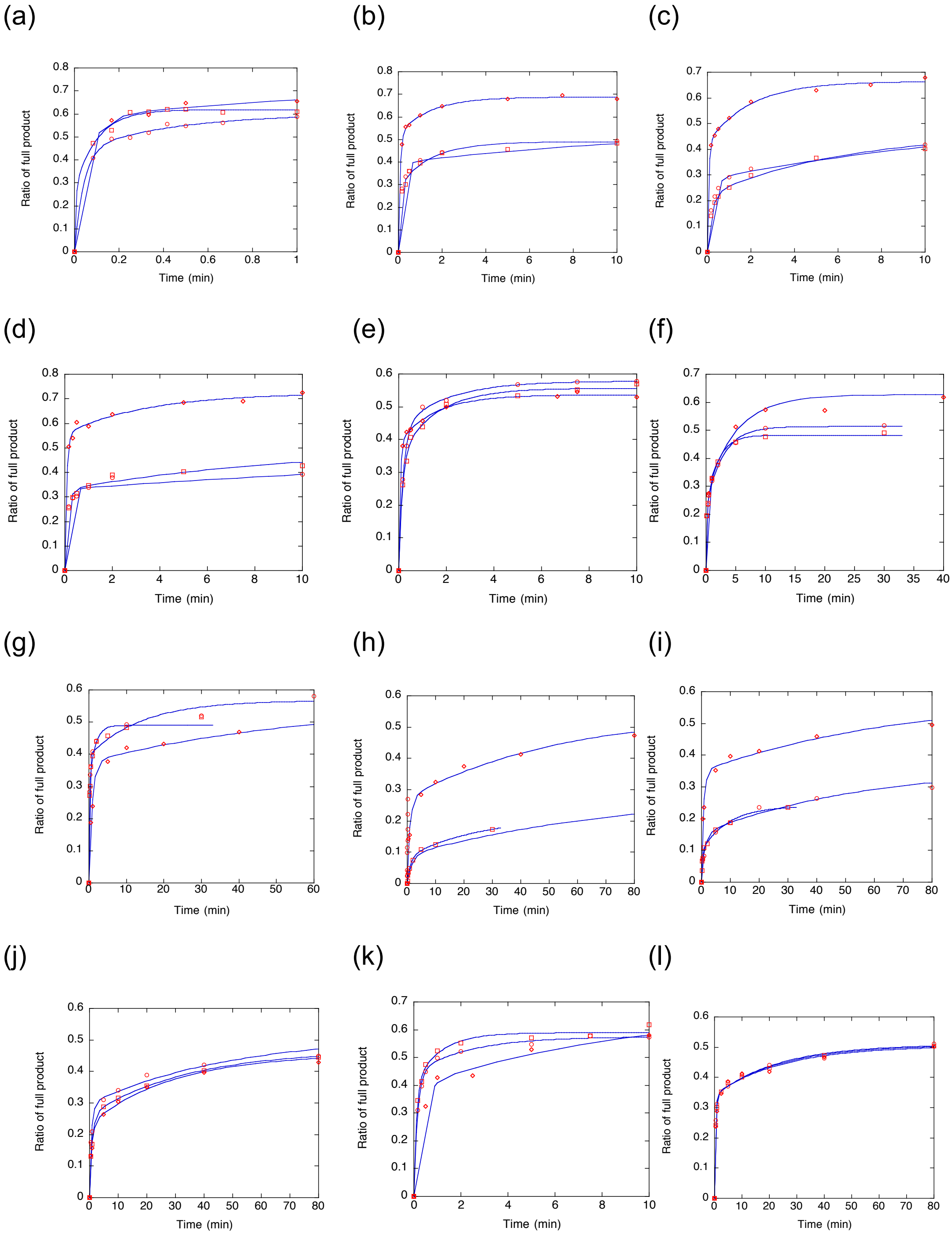
(a)
(b)
(c)
(d)
(e)
(f)
(g)
(h)
(i)
(j)
(k)
(l)
Ratio of full product
Time (min)

(m) (n) (o)

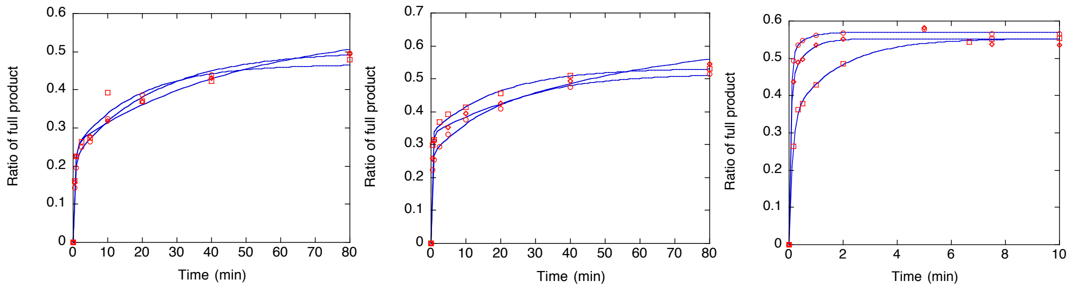

Figure S3. Time course of full-length product of the replication reaction. (a-e) Results in reactions in 1 mM potassium chloride (KCl) without poly(ethylene glycol200) (PEG200) and (a) without ligand, (b) with 10 μM naphthalene diimides (NDI)-DM, (c) with 10 μM cyclic (c)NDI1, (d) with 10 μM cNDI2, and (e) with 50 μM fisetin. (f-j) Results of reactions with 100 mM KCl without PEG200 and (f) without ligand, (g) with 10 μM NDI-DM, (h) with 10 μM cNDI1, (i) with 10 μM cNDI2, and (j) with 50 μM fisetin. (k-o) Results of reactions with 1 mM KCl and 20 wt% PEG200 and (k) without ligand, (l) with 10 μM NDI-DM, (m) with 10 μM cNDI1, (n) with 10 μM cNDI2, and (o) with 50 μM fisetin. The reactions were performed with 1 μM KF and 250 μM deoxynucleoside triphosphates (dNTPs) in the buffer containing 10 mM Tris-hydrochloric acid (HCl) (pH 7.5), 8 mM magnesium chloride ($MgCl_2$), 1 or 100 mM KCl, and 0 or 20 wt% PEG200 at 37 °C. All plots show the results of triplicate reactions in each condition. The dotted lines are fitted to average value of triplicate data.

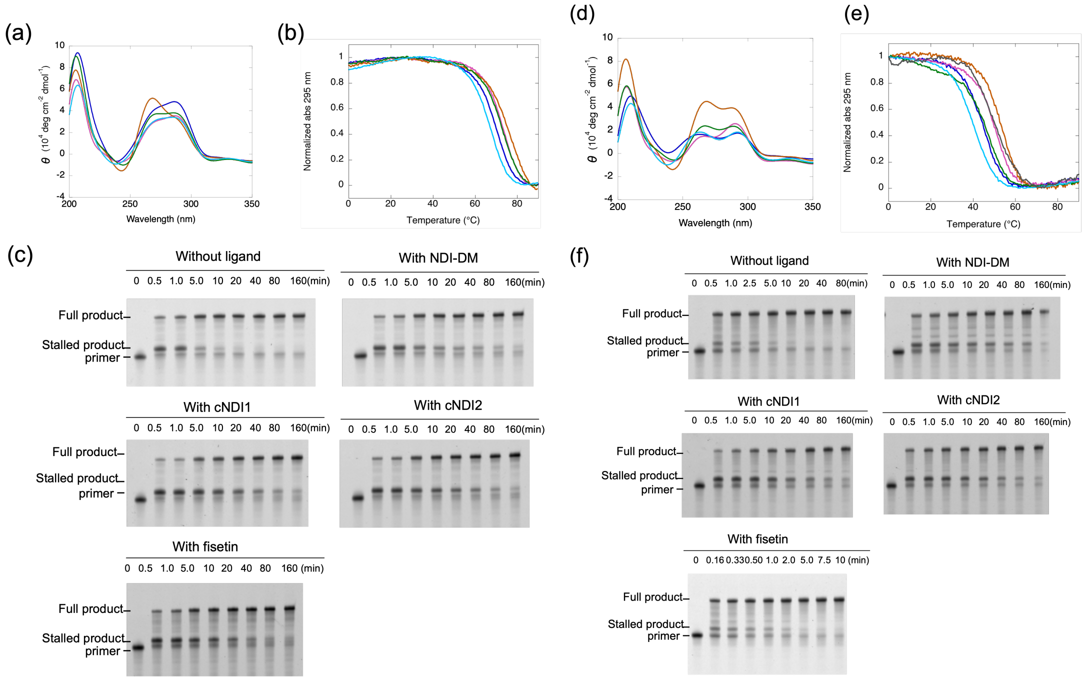

Figure S4. Spectroscopic data and replication results in the presence of 100 mM potassium chloride (KCl) or in the presence of 1 mM KCl and 20 wt% poly(ethylene glycol200) (PEG200) (A) CD spectra of 5 μM $(T_2AG_3)_4$ in the presence of 100 mM KCl without ligand (blue) and with 10 μM naphthalene diimides (NDI)-DM (pink), cNDI1 (brown), cNDI2 (green), or 50 μM fisetin (light blue) at 37 °C. (B) Ultra violet (UV) melting curves of 5 μM $(T_2AG_3)_4$ in the presence of 100 mM KCl without ligand (blue) and with 10 μM NDI-DM (pink), cNDI1 (brown), cNDI2 (green), or 50 μM fisetin (light blue). (C) Polyacrylamide gel electrophoresis (PAGE) images of time courses of the replication of 1 μM $(T_2AG_3)_4$-containing template DNA in the presence of 100 mM KCl in the absence or presence of 10 μM ligands. The reaction was carried out with 1 μM KF exo- and 250 μM deoxynucleoside triphosphates (dNTPs). (D) CD spectra of 5 μM $(T_2AG_3)_4$ in the presence of 20 wt% PEG200 without ligand (blue) and with 10 μM NDI-DM (pink), cNDI1 (brown), cNDI2 (green), or 50 μM fisetin (light blue) at 37 °C. (E) UV melting curves of 5 μM $(T_2AG_3)_4$ in the presence of 20 wt% PEG200 without ligand (blue) and with 10 μM NDI-DM (pink), cNDI1 (brown), cNDI2 (green), or 50 μM fisetin (light blue). (F) PAGE images of time courses of the replication of 1 μM $(T_2AG_3)_4$-containing template DNA in the presence of 20 wt% PEG200 in the absence or presence of 10 μM ligands. The reaction was carried

out with 1 μM KF exo- and 250 μM dNTPs. All experiments were performed in the buffer containing 10 mM Tris-hydrochloric acid (HCl) (pH 7.5) and 8 mM magnesium chloride ($MgCl_2$) at 37 °C.
